# Revolutionizing Breast Surgery: A Case Report on the Excision of a Large Fibroadenoma Suggestive of Phyllodes Tumor Under Thoracic Spinal Anesthesia

**DOI:** 10.7759/cureus.73650

**Published:** 2024-11-13

**Authors:** Richa Chandra, Kartik Sonawane

**Affiliations:** 1 Anesthesiology, Rohilkhand Medical College and Hospital, Bareilly, IND; 2 Anesthesiology, Ganga Medical Centre and Hospitals, Pvt. Ltd, Coimbatore, IND

**Keywords:** benign breast tumor, breast surgery, erector spinae block, fibroadenoma, general anesthesia alternative, respiratory comorbidities, thoracic spinal anesthesia

## Abstract

Fibroadenomas are benign breast tumors, typically affecting middle-aged women, with rapid growth in some cases leading to compression of surrounding structures and presenting a clinical challenge. This case illustrates the successful use of thoracic spinal anesthesia (TSA) for excising a large fibroadenoma with characteristics suggestive of a phyllodes tumor, a procedure traditionally conducted under general anesthesia (GA). The anesthesia was administered using 1.5 ml of 0.5% isobaric levobupivacaine and 5 micrograms of dexmedetomidine, complemented by an ultrasound-guided erector spinae plane block (ESPB) for postoperative analgesia. The patient experienced minimal hemodynamic fluctuations and achieved immediate postoperative mobilization. This case underscores TSA as a safe, effective alternative to GA, particularly beneficial in patients with respiratory considerations, offering stable intraoperative conditions and rapid recovery.

## Introduction

Fibroadenomas are benign breast tumors primarily affecting women aged 15 to 35 [1], with a prevalence of 27.6% in those aged 18 to 40 [2], and an overall incidence of 2.2% in adolescents [3]. Their growth generally slows as women enter their 30s and 40s, yet they may still appear in middle-aged women due to hormonal shifts, lifestyle, and biological variations [4]. Phyllodes tumors, in contrast, are rare fibroepithelial lesions, accounting for just 0.3% to 0.5% of female breast tumors, with an incidence of approximately 2.1 cases per million [5]. These tumors most often occur in women aged 45-49, highlighting their distinct age distribution and rarity compared to fibroadenomas [6,7]. These tumors are rare in men and less common in postmenopausal women. The etiology of fibroadenomas remains unclear, with most cases being slow-growing. However, in some patients, fibroadenomas can exhibit rapid growth, resulting in a significant increase in size. This rapid growth pattern sometimes raises concerns for differentiation from phyllodes tumors, which also grow quickly and carry a potential risk of malignancy. This distinction is crucial, as phyllodes tumors can be cancerous and may require more aggressive management due to their tendency to increase in size.

Phyllodes tumors present a clinical challenge due to their compressive effects on surrounding structures, especially in the breast region. Treatment typically involves wide excision or mastectomy, with general anesthesia (GA) being the standard approach. Unlike routine breast surgeries, excision of a large phyllodes tumor requires wider margins to prevent recurrence, as well as careful consideration of the tumor’s rapid growth and impact on adjacent structures. The tumor’s size can compress the chest wall, often necessitating specialized ventilatory adjustments during surgery. Additionally, reconstructive surgery may be required to maintain breast symmetry, and there is a heightened risk of complications such as hematoma and wound issues, which demand vigilant postoperative management. These factors collectively make phyllodes tumor surgery more complex than typical breast procedures [8].

However, in this case report, we describe a novel approach utilizing thoracic spinal anesthesia (TSA), previously reported for various breast surgeries but, to our knowledge, not for the excision of such a large fibroadenoma with characteristics highly suggestive of a phyllodes tumor. The rapid growth and compressive effects on surrounding structures raised suspicion for phyllodes, making TSA an innovative choice to manage both the surgical and anesthetic complexities of this case. This manuscript adheres to the applicable CARE guidelines. The patient and her family provided written informed consent for the procedure under TSA and for the case report to be published, with her identity kept confidential.

## Case presentation

A 42-year-old female patient of ASA II status presented with a significantly large tumor in her left breast. She reported a sudden increase in the size of the lump over the past three months, to the point where lying supine for extended periods caused a sensation of chest compression due to the tumor’s weight. Additionally, she had a history of well-controlled bronchial asthma, managed with bronchodilators.

Her preoperative vitals and oxygen saturation were within normal limits. Physical examination revealed a large mass visibly compressing her chest, causing discomfort in the supine position due to its size. Preoperative blood investigations, electrocardiogram (ECG), and chest X-ray were within normal limits. Given her medical history and the tumor’s size, the surgical team initially recommended a mastectomy under GA. However, considering the patient’s discomfort and respiratory history, they discussed and agreed on an alternative approach using TSA.

Anesthetic management

After thorough preoperative counseling and ensuring adherence to fasting guidelines, the patient was re-explained about the TSA procedure. Upon arrival in the operating room, a 500 ml Ringer's lactate infusion was initiated, and 4 mg of intravenous ondansetron was administered. Routine intraoperative monitoring was instituted, including ECG, pulse oximetry, and noninvasive blood pressure monitoring. The patient was positioned sitting with her head slightly lowered, and an assistant was instructed to support both shoulders, encouraging the patient to relax.

**Figure 1:**
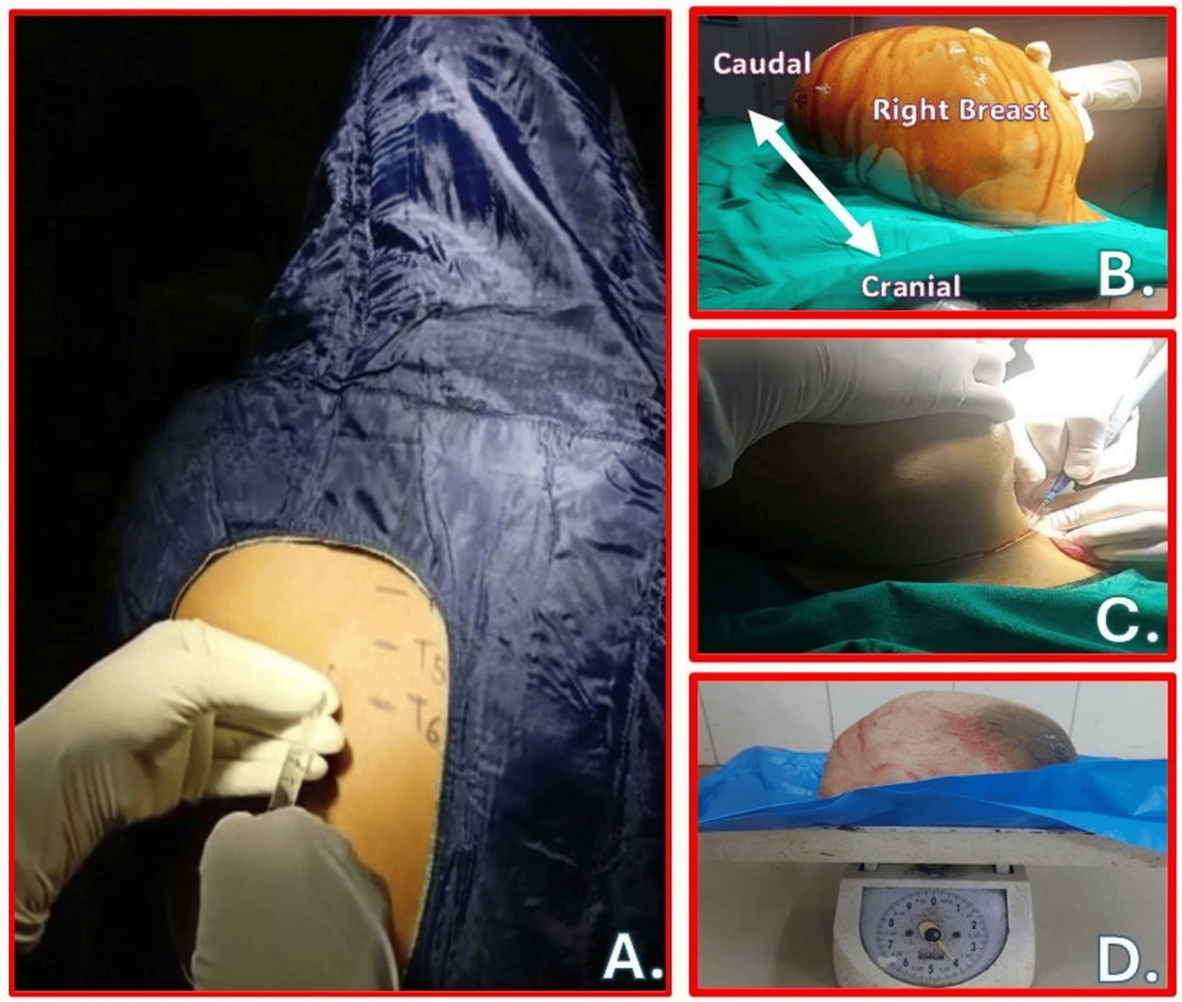
Clinical images of large fibroadenoma excision surgery under thoracic spinal anesthesia (A) Position of patient and needle insertion point between T5-T6 spinal level for thoracic spinal anesthesia. (B) Postanesthesia intraoperative image of large right breast tumor before excision. (C) Surgical incision around breast during excision. (D) Excised tumor and breast tissues Source: This figure was created by the second author (KS)

TSA was administered at the T5-T6 level (Figure 1A), marked beforehand using ultrasound guidance, following the technique described by Chandra et al. [9]. After subcutaneous infiltration with 2 ml of lidocaine and adrenaline, a 26-gauge Quincke spinal needle was inserted. Upon confirming backflow of clear cerebrospinal fluid (CSF), 1.5 ml of 0.5.% isobaric levobupivacaine combined with 5 micrograms of dexmedetomidine was injected to complete the subarachnoid block (SAB). The patient was immediately positioned supine and connected to a supplemental oxygen at 4-6 L/min via a Hudson mask, with end-tidal CO_2_ monitoring initiated.

Following the SAB, sensory blockade extended bilaterally from the C6-C7 level to T8. At the same time, the motor function of the upper extremities remained intact, as indicated by an epidural scaling score for arm movement (ESSAM) of zero (Table 1) [10]. Bilateral lower limb motor function was also preserved, confirming a successful, target-specific block.

**Table 1:** Epidural scaling score for arm movement (ESSAM) grading system

Grades	Movements		
	Hand grip	Wrist flexion	Elbow flexion
	(C8-T1)	(C7-C8)	(C5-C6)
0	Present	Present	Present
1	Absent	Present	Present
2	Absent	Absent	Present
3	Absent	Absent	Absent

Intraoperatively, intravenous fentanyl (75 µg) and midazolam (1 mg) were administered to enhance comfort. One episode of hypotension (95/68 mmHg) occurred but was promptly corrected with an intravenous mephentermine (6 mg). The surgery lasted 50 minutes, with a total blood loss of approximately 350 ml. Immediately following the procedure, an ultrasound-guided erector spinae plane block (ESPB) was performed at the T5 level using 15 ml of 0.25% ropivacaine for additional postoperative analgesia. The excised tumor, measuring 25 × 19 × 13 cm and weighing 4.0 kg, was sent for biopsy (Figure 1D).

Postoperative course

Following surgery, the patient smoothly transferred herself from the operating table to the stretcher, demonstrating rapid recovery from sedation, intact motor function (reconfirmed by ESSAM), and an acceptable comfort level. She was observed in the postanesthesia care unit for one hour, during which her vital signs remained stable. Her pain score was 0, and during the subsequent postoperative period, it ranged from 1 to 3 on the visual analog scale (VAS) of 0 to 10, indicating minimal pain. Approximately 10 hours postoperatively, the patient requested her first dose of rescue analgesia in the form of 50 mg intravenous tramadol. The patient was discharged without complications on the third postoperative day.

**Figure 2:**
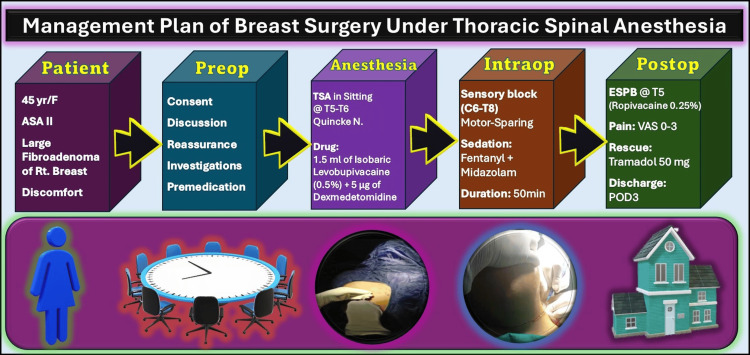
Perioperative management plan of the large fibroadenoma excision surgery under thoracic spinal anesthesia F: Female; ASA: American Society of Anesthesiologists; Rt: right; TSA: thoracic spinal anesthesia; N: needle; ESPB: erector spinae plane block; VAS: visual analog scale; POD: postoperative day Source: This figure was created by the second author (KS)

The complete management plan of this case is depicted in Figure 2, outlining the decision-making process, anesthesia plan, intraoperative strategy, and postoperative care. This figure also highlights the detailed perioperative journey, including the use of TSA as the primary anesthesia modality, the intraoperative measures to ensure patient comfort and safety, and the postoperative analgesia plan, ensuring a smooth and pain-controlled recovery.

## Discussion

TSA, although less commonly used than lumbar spinal anesthesia, has gained attention in recent years as a viable alternative for surgeries traditionally performed under GA. Jonnesco pioneered administering SABs at nonconventional sites [11]. Magnetic resonance imaging studies by Lee et al. [12] later confirmed ample space between the posterior dura mater and the spinal cord, particularly at the thoracic level, with the greatest distance observed at the T5 level. Recent studies in Indian populations by Chandra et al. [13] further support the safety and efficacy of this technique, demonstrating similar findings. Contrary to common belief, there are minimal chances of neurological injury when experienced hands carry out the procedure.

TSA for breast surgeries is a relatively newer modality. Madishetti et al. were the first to utilize this technique in a patient with bronchiectasis undergoing a modified radical mastectomy, using hyperbaric bupivacaine and an additional axillary block for axillary clearance [14]. Subsequently, Elakany et al. demonstrated the efficacy of using isobaric preparations for modified radical mastectomies, finding TSA an effective sole anesthesia modality [15]. Chandra et al. [9] also conducted a study involving 78 patients undergoing modified radical mastectomies under TSA. In their study, all patients exhibited minimal hemodynamic variations, which were easily corrected. Only two patients experienced respiratory discomfort, attributed to medullary hypoperfusion, which resolved with small doses of vasopressors. The British Journal of Anaesthesia recently compiled a comprehensive review of TSA, emphasizing its growing acceptance and establishing it as a significant development in clinical practice [16].

We opted for TSA as the primary anesthesia due to the simplicity of the mastectomy and to avoid potential complications associated with GA. If GA had been chosen, it would have necessitated additional regional analgesia, such as chest wall blocks (pectoralis or serratus anterior blocks, or alternatives like paravertebral block (PVB)) or ESPB. However, anterolateral chest wall blocks were ruled out due to tumor-induced distortion of fascial planes, risking incomplete analgesia. GA would also have required specific ventilatory strategies: unilateral tumor swelling would have necessitated individualized lung ventilation adjustments, such as using a low tidal volume (6-8 mL/kg of ideal body weight) with a slightly increased respiratory rate to prevent overdistension and maintain compliance. Positioning could have compromised lung compliance on one side, making peak and plateau pressure monitoring essential to assess ventilation. A moderate level of positive end-expiratory pressure (e.g., 5 cm H_2_O) would also have been needed to keep alveoli open and counteract any atelectasis from tumor compression. Additionally, close monitoring of end-tidal CO_2_ and oxygen saturation would have been necessary to ensure adequate gas exchange. Preoperative pulmonary function testing was not performed, as the patient displayed normal respiratory status, with discomfort only in the supine position due to the tumor’s size, preferring nonsupine positions during sleep and routine activities. TSA provided a safer, more controlled anesthesia environment. For postoperative analgesia, ESPB was chosen for its reliable, prolonged pain relief. Additionally, we selected TSA over PVB for the anesthesia itself to ensure a complete, target-specific blockade, which was crucial in this case. Our decision was also guided by previous successful outcomes using TSA in similar procedures, reinforcing its efficacy and safety.

At the thoracic level, the relatively lower CSF volume and thinner spinal nerves allowed for a faster and more predictable onset of anesthesia. A minimal local anesthetic dose at the target site helped prevent significant hemodynamic disturbances. An assistant provided manual support to the swelling (Figures 1B-1C), alleviating external compression and ensuring that the patient remained comfortable in the supine position throughout the surgery. It helped prevent discomfort and allow for optimal surgical conditions. With both upper and lower extremities spared, the patient could mobilize immediately after the surgery. The addition of an ESPB postoperatively ensured prolonged pain relief, with the patient requiring minimal rescue analgesia in the first 24 hours postsurgery. This multimodal approach to anesthesia and analgesia significantly enhanced the patient’s recovery, allowing for early ambulation and discharge.

## Conclusions

This case highlights the successful use of TSA for excising a large fibroadenoma with characteristics suggestive of a phyllodes tumor, a procedure traditionally performed under GA. TSA provided optimal intraoperative conditions, stable hemodynamics, and rapid postoperative recovery, underscoring its potential as a valuable alternative in complex cases. Given the possibility of phyllodes, TSA allowed for precise anesthetic control while accommodating the surgical demands of a large, possibly malignant mass, demonstrating its efficacy and safety in such challenging scenarios.

With growing familiarity and expertise in TSA’s technique and hemodynamic management, it shows promise for patients at high risk of respiratory complications associated with GA. However, for patients with significant cardiac risk, where hemodynamic stability is crucial, thoracic PVB may be a safer alternative, providing adequate surgical anesthesia and postoperative analgesia with or without adjunctive blocks. As evidence supporting the safety and efficacy of TSA continues to grow, it is emerging as a valuable tool in the anesthesiologist’s repertoire, particularly for surgeries involving the thoracic region and patients with respiratory or cardiovascular comorbidities.
